# Effects of wavelength and spinal cord injury segment on photobiomodulation efficacy: Monte Carlo simulation in a rat model

**DOI:** 10.1117/1.JBO.30.S2.S23907

**Published:** 2025-08-04

**Authors:** Zemeng Chen, Faguang Wang, Chenxi Yang, Houqing Long, Ting Li

**Affiliations:** a Chinese Academy of Medical Sciences, Biomedical Engineering Institute, Peking Union Medical College, Tianjin, China; b Shenzhen People’s Hospital, Division of Spine, Department of Orthopedic Surgery, Shenzhen, China

**Keywords:** spinal cord injury, photobiomodulation, Monte Carlo simulation, photon migration, injured spinal level

## Abstract

**Significance:**

Spinal cord injury (SCI) is a severe condition characterized by complex pathophysiology and challenges in neural regeneration within the central nervous system, presenting substantial difficulties for repair. Although surgery and medication are employed for early rehabilitation and complications prevention, biomaterial transplantation, stem cell therapy, and neuromodulation techniques have been extensively investigated to promote axon regeneration and neural circuit remodeling in SCI, though results remain suboptimal.

**Aim:**

Photobiomodulation (PBM), with its strong anti-inflammatory and tissue repair effects, is gaining increasing attention as a noninvasive physical therapy. However, only a limited number of studies have focused on injury site and wavelength selection. This study addresses these issues.

**Approach:**

To tackle this issue, we performed low-cost and quantitative comparisons of light distribution in a SCI rat model using Monte Carlo simulations of light propagation. The SCI models encompassed cervical (C2, C4, and C6) and thoracic (T1, T3, T7, and T10) spinal regions, and simulations were performed for four wavelengths (660, 808, 980, and 1064 nm).

**Results:**

The cervical spinal injuries benefit more from PBM than thoracic spinal injuries due to higher photon fluence in the cervical spinal cord compared with the thoracic region. Notably, 1064 nm demonstrated deeper penetration than 980, 808, and 660 nm.

**Conclusions:**

We present a robust computational framework and empirical insights to inform the optimization of PBM parameters for SCI treatment. Our simulation and comparisons offer valuable reference for researchers and clinicians in performing precise and quantitative PBM treatment for SCI. As further studies are conducted, we aim to develop standardized, personalized optical parameters for clinical PBM in SCI treatment.

## 1 Introduction

Spinal cord injury (SCI) is a significant disease burden and a severe public health issue due to its extremely high incidence of disability, serious complications, and substantial medical costs.^1^^–^^4^ SCI is classified into acute and chronic phases: the acute phase occurs within hours to a few weeks after the injury, whereas the chronic phase lasts from months to years post-injury.^5^^–^^7^ Early decompression within 8 h after traumatic SCI may improve functional and neurological outcomes in affected individuals. However, surgery cannot prevent secondary damage from spinal cord necrosis and hemorrhage, which are major factors hindering the repair of central nervous system injuries.^8^^–^^11^ In the early stages of SCI, high-dose methylprednisolone was believed to positively affect neural repair, but its use is associated with severe complications, including infection, respiratory damage, gastrointestinal bleeding, and even death.^12^^,^^13^ Biomaterial transplantation and stem cell therapy have been shown to aid neuro remodeling, protection, and structural repair; however, acute SCI may not be suitable for direct cell transplantation into the injured area.^14^^,^^15^ Physical therapies such as functional electrical stimulation, low-intensity pulsed ultrasound, magnetic stimulation, and photobiomodulation [PBM, or low-level therapy (LLL)] have recently been found to regulate nerve function during recovery from SCI.^16^^,^^17^ Compared with neuromodulation devices, PBM offers an affordable, noninvasive, and low-cost alternative that can be effective in treating central nervous system damage.^10^^,^^18^

Several studies have demonstrated that PBM promotes functional recovery by reducing neuroinflammation and enhancing neuronal axon regeneration after SCI.^19^ PBM therapy at 810 nm has been reported to upregulate macrophage secretion of neurotrophic factors via PKA-CREB, promoting neuronal axon regeneration *in vitro* while inhibiting astrocyte activation and secretory function through changes in macrophage polarization.^20^ LED red light (670 nm) significantly reduced hypersensitivity following mild T10 hemi-contusion SCI in rats, accompanied by improved dorsal column pathway functional integrity and locomotor recovery.^21^ Another study confirmed that PBM at 660 nm alleviated mechanical and heat hyperalgesia in T13 compression SCI rats and reduced interleukin-6 expression to mitigate inflammation caused by SCI.^22^ However, no consensus has been reached on the most effective treatment parameters, such as wavelengths and injury segments, for both animals and humans.

Computing the fluence distribution in SCI rats enhances the understanding of PBM mechanisms and aids in its optimization.^23^ Since Wilson and Adam first introduced the Monte Carlo simulation to laser-tissue interactions, it has undergone several improvements, making it a reliable, precise, and flexible method for comparing and optimizing instrument and experimental designs.^24^^–^^27^ A previous study provided a detailed description of implementing a Monte Carlo model for photon migration in voxelized media (MCVM).^28^ The MCVM can serve as a reliable tool for simulations in fully heterogeneous three-dimensional (3D) models, enabling its applications in simulating photon migration in complex, realistic models of biological tissues, such as those obtained by MRI, CT, or other imaging techniques.^29^ A high-resolution atlas of the adult rat, consisting of 9475 cryosection images created using serial cryosection-milling techniques, provides an extremely accurate anatomical structure of the entire rat body, particularly the spinal cord, for use with MCVM.^30^

In this study, we utilized the MCVM and rat atlas to simulate the photon distribution of PBM at four different wavelengths (i.e., 660, 808, 980, and 1064 nm) across seven SCI segments (i.e., C2, C4, C6, T1, T3, T7, and T10) in the rat cervical and thoracic spine. The results included photon fluence, absorption, and scattering at varying depths and tissues. The results indicated that cervical spinal injuries may benefit more from light therapy than thoracic spinal injuries as the photon fluence at the cervical spinal cord was higher than that at the thoracic spine. Surprisingly, the best treatment effect may be achieved with 1064 nm light as it provides greater penetration depth and photon fluence to the spinal cord. Based on the comparison of photon distribution in SCI rat tissues at different injury levels and wavelengths, the treatment parameters for PBM in animal experiments can be optimized. We believe this study provides valuable insights for developing treatment parameters for quantitative, precise, and personalized phototherapy of SCI.

## 2 Method

### 2.1 Spinal Cord Injury Rat Model

**Fig. 1:**
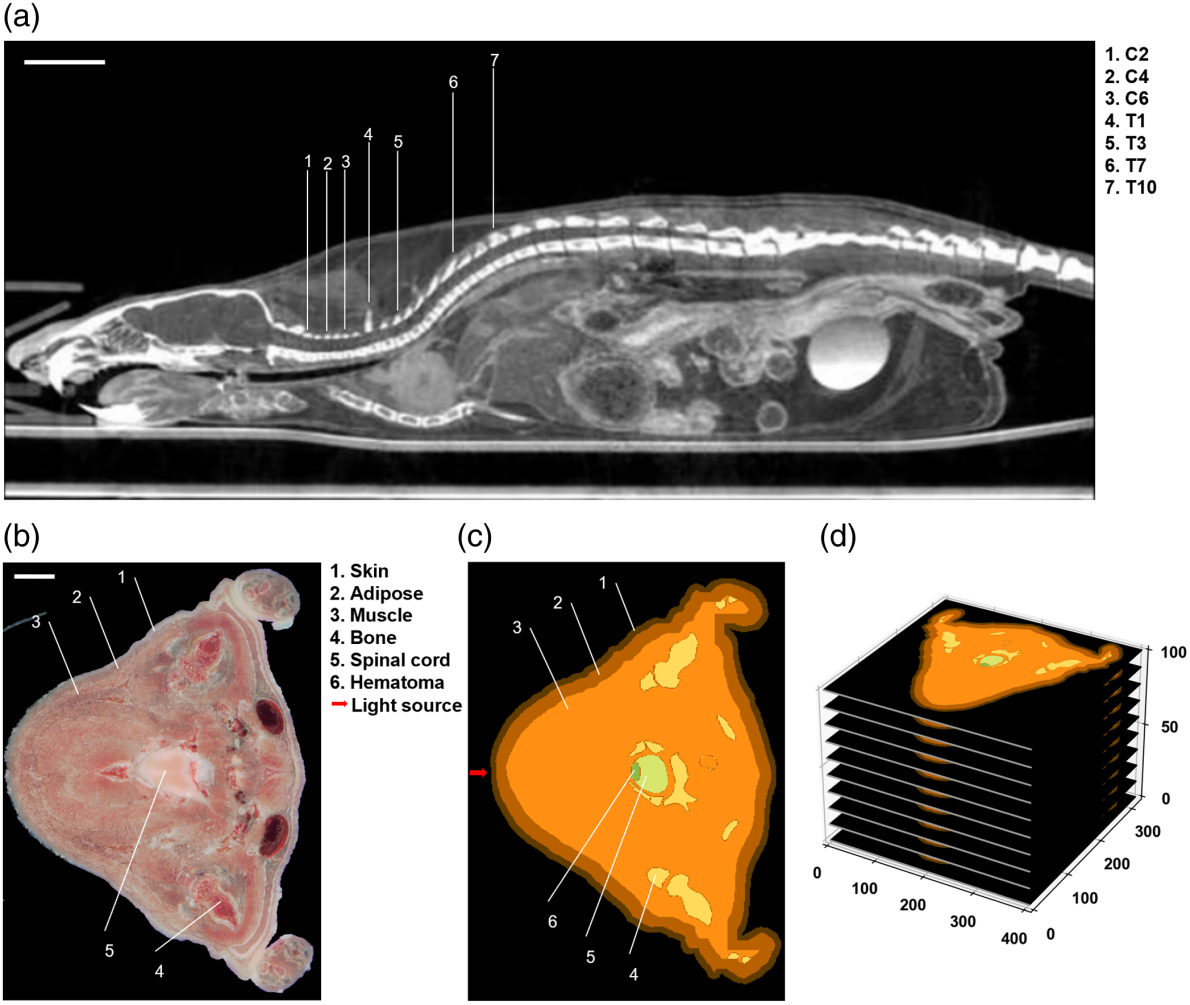
SD rat atlas and illustration of Monte Carlo simulation. (a) Sagittal section of the rat model.^31^ Scale bar = 10 mm. (b), (c) The cross section and model for the Monte Carlo simulation of C2, and a cross section including the stomach, please refer to the Supplementary Material. Scale bar = 5 mm. (d) The three-dimensional media matrix of C2.

This study employed a high-resolution atlas of the adult rat to construct a light propagation model for the SCI rat.^30^ The Sprague–Dawley (SD) rat anatomical atlas was generated using serial cryosection-milling and digital imaging techniques. The entire rat was transversely sectioned and imaged at 0.2 mm intervals, with a high resolution of 0.1*0.1  mm. Anatomical structures, arterial vessels, and skeletal systems were segmented using an auto-segmentation program, whereas most internal organs were manually segmented by tracing digital images from these serial cryosection slices under the guidance of an experienced anatomist. For the simulation, the cervical and thoracic spines of the rat atlas were extracted to construct the SCI model [Fig. 1(a)]. The SCI model was modified manually by removing the spinous processes of C2, C4, C6, T1, T3, T7, and T10, and a hematoma was implanted in the spinal cord. The hematoma was assumed to be an ellipsoid-shaped object with a volume of $4\;{\mathrm{mm}}^{3}$ (coronal section: 1 mm, median sagittal section: 2 mm, transverse section: 2 mm). The hematoma was caused by spinal cord edema resulting from slowed blood circulation after bone deformation, which led to bone marrow damage and bleeding. Therefore, its optical parameters were approximated to those of blood. Taking C2 as an example, the model and tissue division used for the Monte Carlo simulation were detailed, and a Gaussian light source was incident along the sagittal axis with the projection point at the center of the skin surface injury, as shown in Figs. 1(b) and 1(c). Figure 1(d) illustrates the schematic of the 3D stacking of segmented images used in the simulation. The optical properties of each tissue at four wavelengths are presented in Table 1.

**Table 1:** Optical properties of SCI rats’ tissue for 660, 808, 980, and 1064 nm.

Type	660 nm				808 nm				980 nm				1064 nm			
	$\mu_{a}/{\mathrm{cm}}^{-1}$	$\mu_{s}/{\mathrm{cm}}^{-1}$	n	g	$\mu_{a}/{\mathrm{cm}}^{-1}$	$\mu_{s}/{\mathrm{cm}}^{-1}$	n	g	$\mu_{a}/{\mathrm{cm}}^{-1}$	$\mu_{s}/{\mathrm{cm}}^{-1}$	n	g	$\mu_{a}/{\mathrm{cm}}^{-1}$	$\mu_{s}/{\mathrm{cm}}^{-1}$	n	g
Skin^32^	0.59	91.55	1.42	0.74	0.43	98.50	1.42	0.80	0.27	107.11	1.42	0.84	0.22	110.07	1.42	0.85
Adipose^32^^,^^33^	1.03	199.00	1.44	0.90	1.03	177.10	1.44	0.90	1.17	177.89	1.44	0.91	1.07	172.11	1.44	0.91
Muscle^32^^,^^33^	0.64	72.40	1.37	0.95	0.39	51.80	1.37	0.95	0.42	53.00	1.37	0.96	0.15	45.00	1.37	0.96
Bone^34^	0.42	207.70	1.56	0.90	0.25	188.90	1.56	0.90	0.28	152.30	1.56	0.90	0.24	148.10	1.56	0.90
Spinal cord^35^^,^^36^	2.14	116.32	1.37	0.90	0.55	102.40	1.37	0.90	0.39	96.68	1.37	0.90	0.31	86.10	1.37	0.90
Blood^36^^,^^37^	1.41	913.61	1.37	0.98	3.36	816.05	1.37	0.98	6.87	718.90	1.37	0.98	5.18	686.96	1.37	0.98
Liver^38^	7.99	67.82	1.38	0.74	5.14	59.27	1.37	0.78	4.76	52.28	1.37	0.79	4.58	50.18	1.37	0.78
Stomach^39^	0.82	47.66	1.45	0.69	0.79	47.03	1.45	0.77	1.14	42.50	1.45	0.79	1.09	43.19	1.45	0.81

$\mu_{a}$ is the absorption coefficient. $\mu_{s}$ is the scattering coefficient. n is the refractive index. g is the scattering anisotropy factor.

### 2.2 MCVM

In this study, we employed the Monte Carlo simulation to model light propagation in the rat spinal cord. The Monte Carlo software used was specifically designed for 3D voxelized media (MCVM), which was independently developed by our team. The algorithm was described in a previous study.^40^ In simple terms, 3D structures with different properties are represented as voxels, and MCVM simulates how photons are randomly scattered or absorbed by these voxels until they either escape or are absorbed. Therefore, as long as the absorption and scattering coefficients of each voxel are accurately measured, MCVM can precisely simulate photon absorption and scattering in tissues. Importantly, the software has no limitations regarding light wavelength, incident direction, source position, or the geometry of the media, making MCVM particularly suitable for simulation photon distribution in the SCI rat atlas. Using MCVM, we can simulate the detailed transmission pathways of photons and generate 3D photon distribution maps within tissues for further analysis.

### 2.3 Simulation and Statistical Analysis

Using MCVM and the model of SCI rat, we evaluated the photon distribution of four different wavelengths (i.e., 660, 808, 980, and 1064 nm) across seven SCI sites (C2, C4, C6, T1, T3, T7, and T10), as shown in Fig. 1(a). The wavelengths of 660, 808, and 980 nm were selected due to their widespread use and demonstrated therapeutic efficacy in previous PBM studies on SCI. The 1064 nm wavelength, commonly applied in light therapy for other conditions such as Alzheimer’s disease, was included to assess its tissue penetration characteristics in comparison. The selected injury sites represent commonly used levels in PBM-SCI animal studies and span a broad range of spinal cord-to-skin distances. The lumbar spine was excluded because the spinal cord in this region disperses into the cauda equina, making it difficult to model accurately. Together, the chosen sites encompass both long and short spinal cord-to-skin distances. A Gaussian light source with a 1.6 cm diameter ($∼2\;{\mathrm{cm}}^{2}$) was applied to each injury site and wavelength for consistency. In all simulations, the initial photon number was set to $10^{7}$ and normalized to the same power (1 W) to ensure comparability across conditions.

According to the results of the Monte Carlo simulation, photon absorption in each voxel and the proportion of spillover photons were recorded. To accurately calculate the photon fluence within each voxel, we applied a precise computational method: the photon absorption value of each voxel was divided by the absorption coefficient of the corresponding tissue type. Using this approach, photon fluence distributions were visualized for all seven SCI segments across the four tested wavelengths. For each tissue type traversed by the photons, the total photon fluence and absorption were calculated by summing the respective values across all constituent voxels. The resulting ratios were then analyzed. In addition, we computed three key optical parameters for each SCI segment and wavelength: the absorbed fraction ratio (A), diffuse reflectance to total transmittance ratio (R), and specular reflectance (S), based on the following definitions. Specifically,

$$A=\frac{\overset{Nz-1}{\underset{iz=0}{\sum }}\overset{Ny-1}{\underset{iy=0}{\sum }}\overset{Nx-1}{\underset{ix=0}{\sum }}A_{xyz}[ix,iy,iz]}{N},$$ (1)

where $N_{x}$, $N_{y}$, and $N_{z}$ represent the dimensions of the model in the x, y, and z directions, respectively; $A_{xyz}$ [ix,iy,iz] denotes the photon absorption at the voxel indexed by $\left(i_{x},i_{y},i_{z}\right)$; and N is the total number of photons input into the model.

$$R=\frac{\overset{Nz-1}{\underset{iz=0}{\sum }}\overset{Ny-1}{\underset{iy=0}{\sum }}\overset{Nx-1}{\underset{ix=0}{\sum }}R_{xyz}[ix,iy,iz]}{N},$$ (2)

$R_{xyz}$ [ix,iy,iz] represents the ratio of diffuse reflectance to total transmittance at the voxel indexed by $\left(i_{x},i_{y},i_{z}\right)$.

S=1−A−R. (3)

When the power of the input light source was set to 1 W, the units of both photon fluence and absorption were expressed at $W/{\mathrm{cm}}^{2}$. As the power of the light source increased, photon fluence and absorption values increased proportionally, consistent with the scaling of the input power. This linear relationship is a result of principles underlying the MCVM used in this study. For the detailed theoretical derivation and methodology, please refer to a previous study.^41^

## 3 Results

### 3.1 Differences in the Penetration Depth and Photon Distribution

**Fig. 2:**
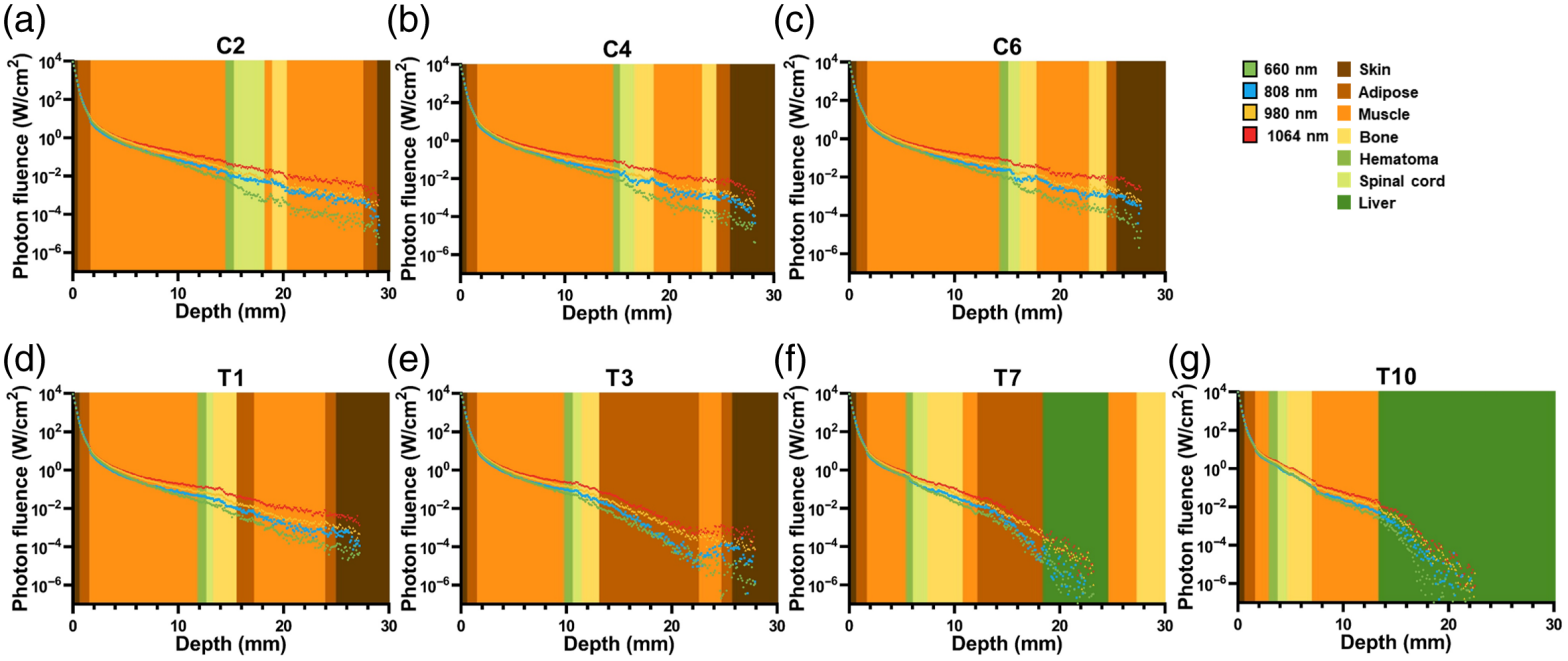
Photon fluence variations following with tissue depth. (a)–(g) The photon fluence variations followed with tissue depth in seven tissues (skin, adipose, muscle, bone, hematoma, spinal cord, and liver) when the light sources were located on C2 (a), C4 (b), C6 (c), T1 (d), T3 (e), T7 (f), and T10 (g).

In the Monte Carlo simulations, photon fluence attenuation curves were plotted for all four wavelengths across the seven SCI segment models. These curves were generated in the cross-sectional plane at the injury center and along the sagittal axis, which is the direction of light propagation, overlaid on the corresponding tissue structures (Fig. 2).

According to the pseudo-color maps, the light sources, all set to 1W, can penetrate superficial tissues and reach the spinal cord and hematoma located at the center of the injury segments. After passing through the skin and adipose tissue, the remaining photon fluence was approximately on the order of $10\;W/{\mathrm{cm}}^{2}$. Among the thoracic segments, T10 is the closest to the skin surface, and when light reaches the SCI center, the photon fluence remains above $1\;W/{\mathrm{cm}}^{2}$. For the other thoracic (T1, T3, and T7) and cervical segments (C2, C4, and C6), the distance between the injury center and the skin gradually increases. As shown in the attenuation curves, photon fluence at these segments decreases to the range of $10^{-1}\;W/{\mathrm{cm}}^{2}$ for T7, T3, and T1 and $10^{-2}\;W/{\mathrm{cm}}^{2}$ for C6, C4, and C2.

When comparing the four wavelengths, light with a wavelength of 1064 nm exhibited the slowest attenuation upon entering the model, followed by 980, 808, and 660 nm, with 808 and 980 nm showing similar attenuation rates. Attenuation in skin and adipose tissue was roughly comparable across all wavelengths, with significant differences observed primarily in the muscle tissue. Except for the T7 and T10 segments, the photon fluence at the lesion center for 1064 nm light was nearly 10 times higher than that for 660 nm light. The discrepancy was more pronounced in the cervical spine. Due to the differing optical properties of these tissues, transitions between tissue types, such as from muscle to bone, resulted in notable changes in photon fluence.

**Fig. 3:**
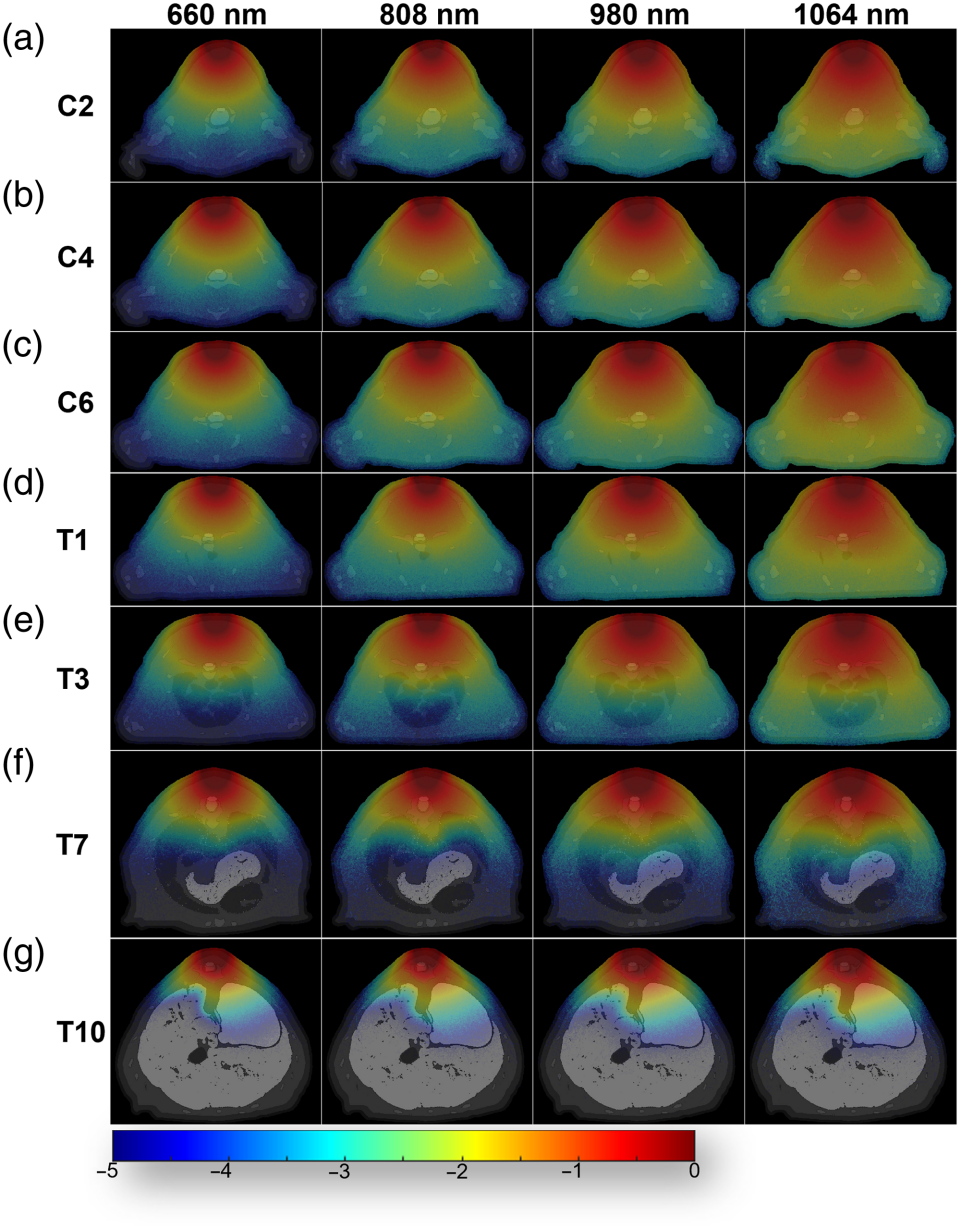
Photon fluence distribution at seven SCI segments. (a)–(g) The photon fluence distribution with the corresponding cross-sectional model as background.

Figure 3 illustrates the photon distribution in the cross-section where the SCI center is located. From the distribution of light sources at four wavelengths across the seven injury centers, it is evident that, in the thoracic segments, particularly T10, the light intensity at the SCI center was higher than in the cervical segments. Light with a wavelength of 1064 nm exhibited the least attenuation as it propagated to the injury center, followed by 980, 808, and 660 nm, which showed the greatest attenuation. This trend was consistent across all seven SCI sites, with the difference in attenuation being more pronounced in the cervical segment than in the thoracic segment.

### 3.2 Differences in Total Photon Fluence Between Tissues

**Fig. 4:**
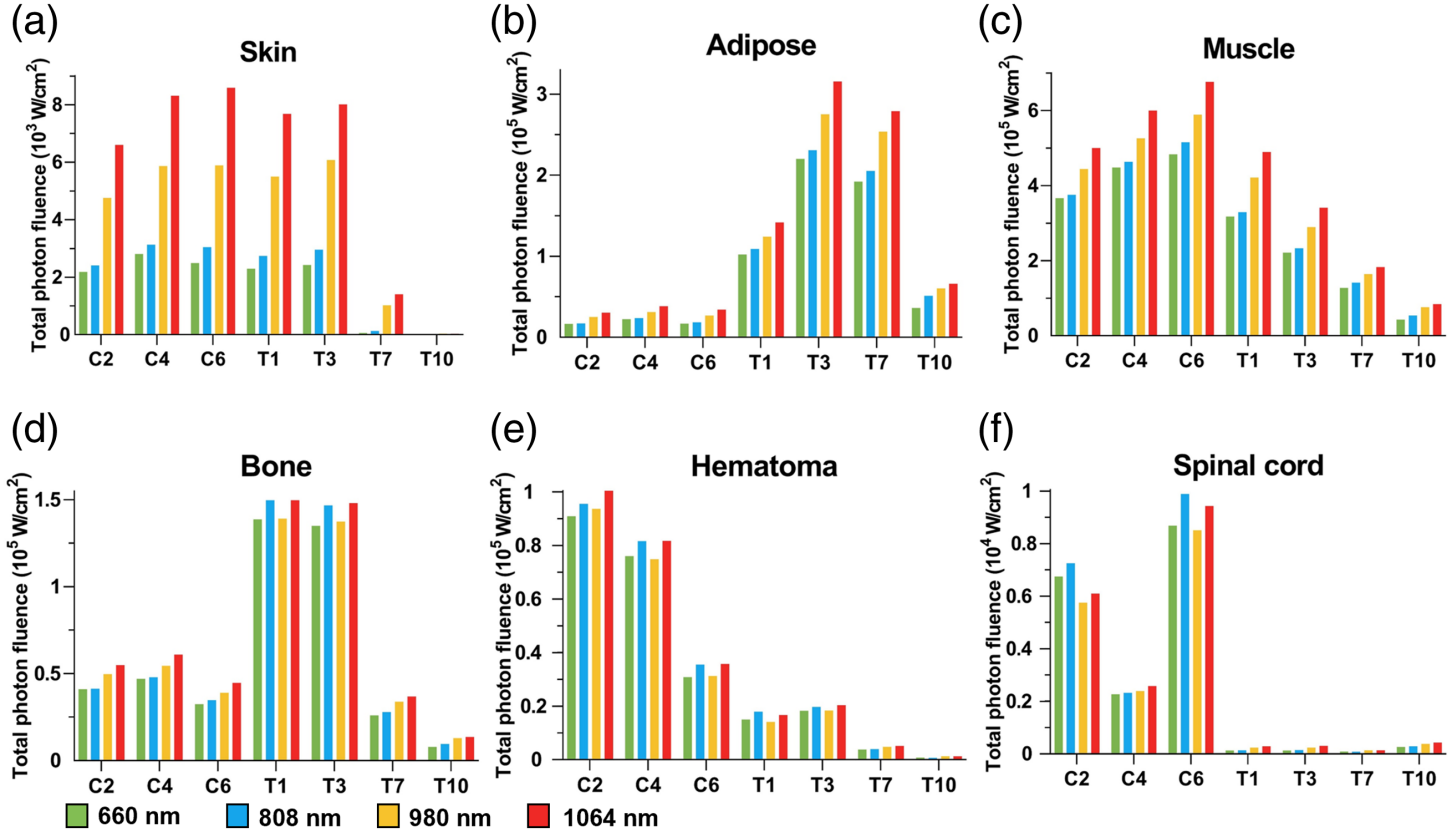
Total photon fluence within different tissues. (a) Skin, (b) adipose, (c) muscle, (d) bone, (e) hematoma, and (f) spinal cord.

Although the penetration depth and coverage area of light determine the extent of PBM’s influence on SCI, achieving sufficient photon fluence to induce photobiological effects remains essential. Therefore, the total photon fluence in various tissues—including skin, adipose, muscle, bone, hematoma, and spinal cord—was calculated and compared across the seven injury sites and four wavelengths (Fig. 4).

In most spinal segments, the total photon fluence in the skin, adipose tissue, muscle, and bone was higher than that in the hematoma and spinal cord, reaching magnitudes of $∼10^{5}$ and $10^{4}\;W/{\mathrm{cm}}^{2}$, respectively. In adipose tissue, the photon fluence was significantly lower in the cervical spine than in the thoracic spine, whereas in hematoma and spinal cord, the trend was reversed. This result highlighted that, although the thoracic spinal cord is anatomically closer to the skin and receives higher light intensity, its photon absorption was less than half that of the cervical spine. Among the cervical levels, C6 exhibited the highest total photon fluence in the spinal cord—$∼10^{4}\;w/{\mathrm{cm}}^{2}$—followed by C2 and C4. In the thoracic segments, total photon fluence in the spinal cord showed less variation. T10 had the highest value at $∼4*10^{3}\;W/{\mathrm{cm}}^{2}$, followed by similar values at T1 and T3, whereas T7 was the lowest at around $1*10^{3}\;W/{\mathrm{cm}}^{2}$.

The total photon fluence across different tissues in the seven injury centers varied significantly depending on the wavelength. In most tissues, light at 1064 nm produced the highest or near-highest photon fluence, followed by 980 and 808 nm, whereas 660 nm generally yielded the lowest. In spinal cord and hematoma tissues, the total photon fluence among the four wavelengths was generally comparable, except in the C2 and C6 spinal cord segments. In these segments, the rank order of photon fluence was 808  nm>660  nm>1064  nm>980  nm.

### 3.3 Differences in the Proportion of Photon Fluence Between Tissues

**Fig. 5:**
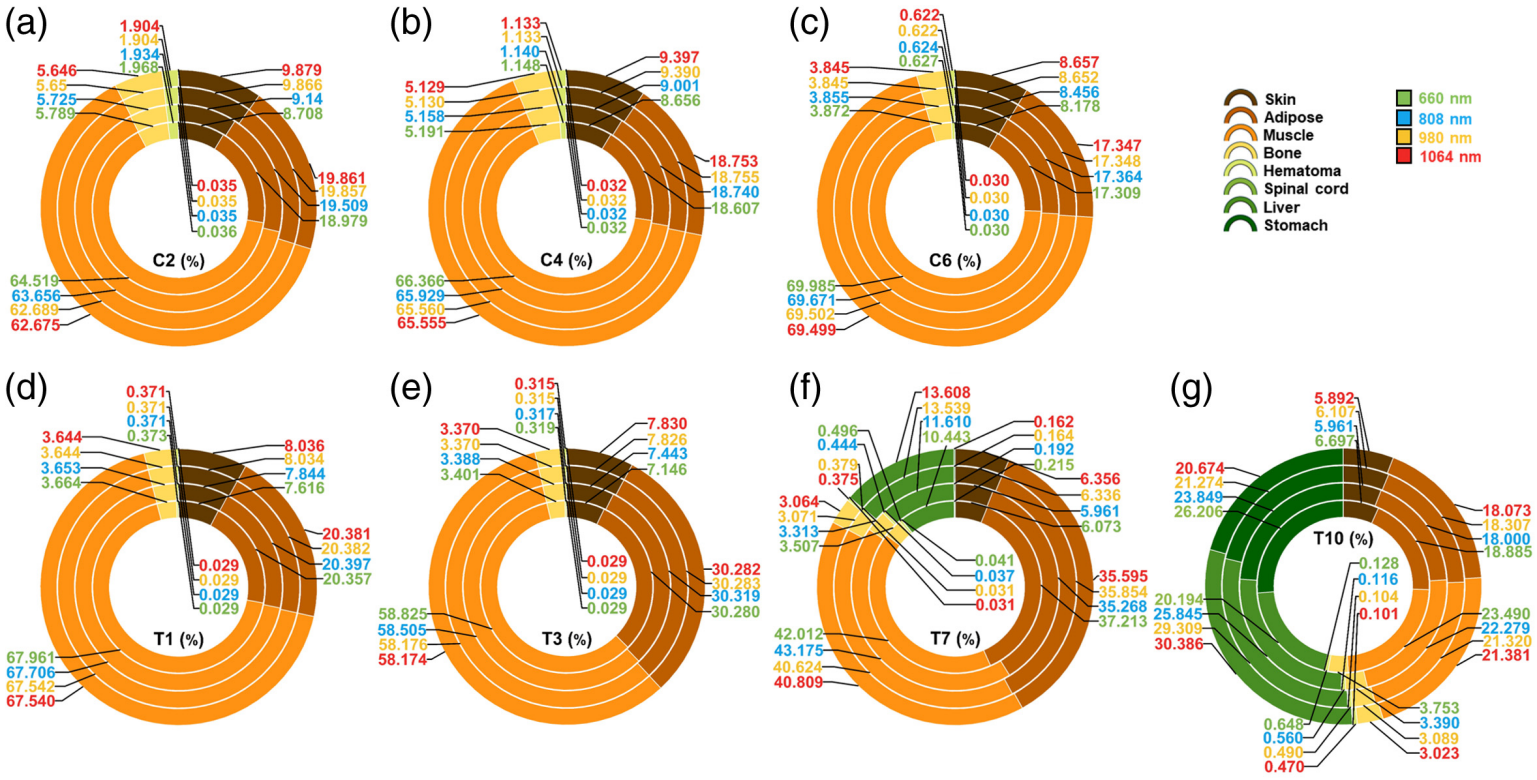
Photon fluence proportion within different tissues. (a)–(e) The model for these five sections (C2, C4, C6, T1, and T3, respectively) contains six types of tissues (skin, adipose, muscle, bone, hematoma, and spinal cord). (f), (g) The model for these two sections (T7 and T10) contains two more tissues (liver and stomach).

To compare the relative contribution of each tissue to the total photon fluence within the injured segment, the photon fluence values for individual tissues were quantified and visualized using donut plots (Fig. 5).

The distribution of photon fluence across tissue types varied substantially. In the C2 to T3 segments, skin, adipose, and muscle collectively accounted for ∼95% of the total photon fluence. This proportion decreased to around 85% in the T7 segment—likely due to the presence of additional abdominal organs such as the liver and stomach—and dropped further to below 45% in the T10 segment. The photon fluence contribution of bone remained relatively consistent across all segments, ranging from 3% to 5%. The hematoma at the C2 level exhibited the highest relative photon fluence among all segments, accounting for ∼2%, which gradually declined along the rostro-caudal axis to ∼0.5% at T10. For the spinal cord, the relative photon fluence showed a gradual decrease and stabilization from C2 to T7. However, a distinct increase was observed at T10, where the value rose from ∼0.035% to 0.041% in earlier segments to 0.128%.

The variation in photon fluence distribution across the four wavelengths was less pronounced than the variation observed across the seven injury sites. Among all tissues, muscle exhibited the largest difference between wavelengths, with a maximum variation of ∼2%. By contrast, differences in other tissues were typically around 1% and were as low as 0.05% in the hematoma and 0.005% in the spinal cord. In terms of relative photon fluence within the spinal cord, light at 660 nm generally accounted for the highest proportion, followed by 808, 980, and 1064 nm.

### 3.4 Differences in Total Photon Absorption Between Tissues

**Fig. 6:**
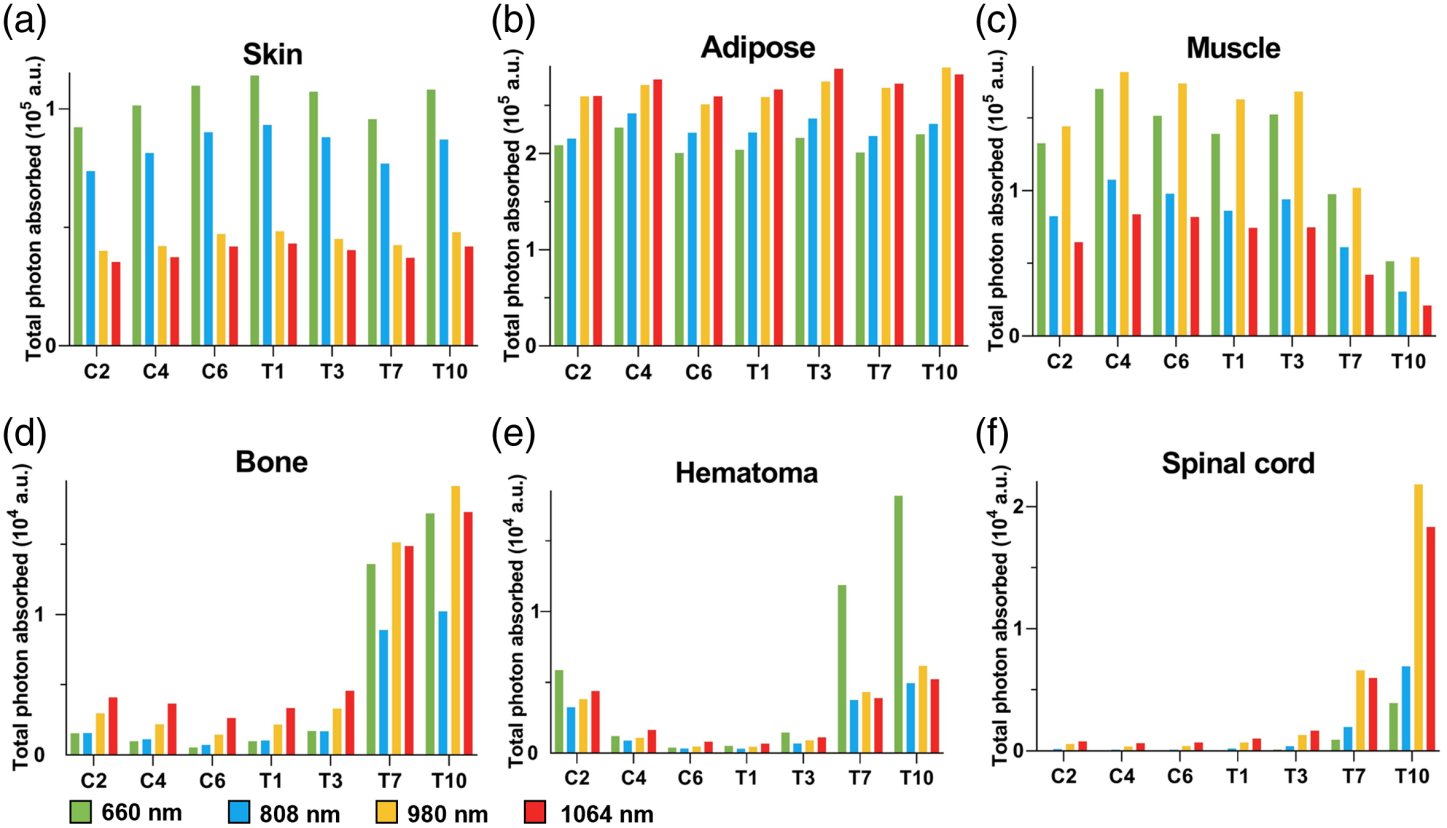
Total photon absorption within different tissues. (a) Skin, (b) adipose, (c) muscle, (d) bone, (e) hematoma, and (f) spinal cord.

When light interacts with biological tissue, it induces not only photobiological effects but also thermal effects, which are represented in the MCVM as light absorption. In this study, we calculated the total photon absorption in various tissues—including skin, adipose, muscle, bone, hematoma, and spinal cord—across seven injury sites and four wavelengths. These values were compared and analyzed (Fig. 6).

Skin, adipose tissue, and muscle exhibited the highest levels of light absorption, followed by bone, with hematoma and spinal cord absorbing the least. Across spinal segments, photon absorption in the skin, adipose, and muscle remained relatively consistent. By contrast, bone, hematoma, and spinal cord showed higher light absorption in the thoracic spine compared with the cervical spine. Among the four wavelengths, 980 nm light resulted in the highest light absorption in adipose and muscle, whereas in skin, it showed the lowest absorption. In the T10 segment, 660 and 980 nm light produced the greatest absorption in hematoma and spinal cord tissues, respectively, whereas the lowest absorption occurred in the C4 segment. For most of the spinal segments, photon absorption in the spinal cord was comparable between 808 and 1064 nm light, whereas greater variability was observed with 660 and 980 nm.

### 3.5 Differences in Photon Proportions among Reflection, Absorption, and Scattering

**Fig. 7:**
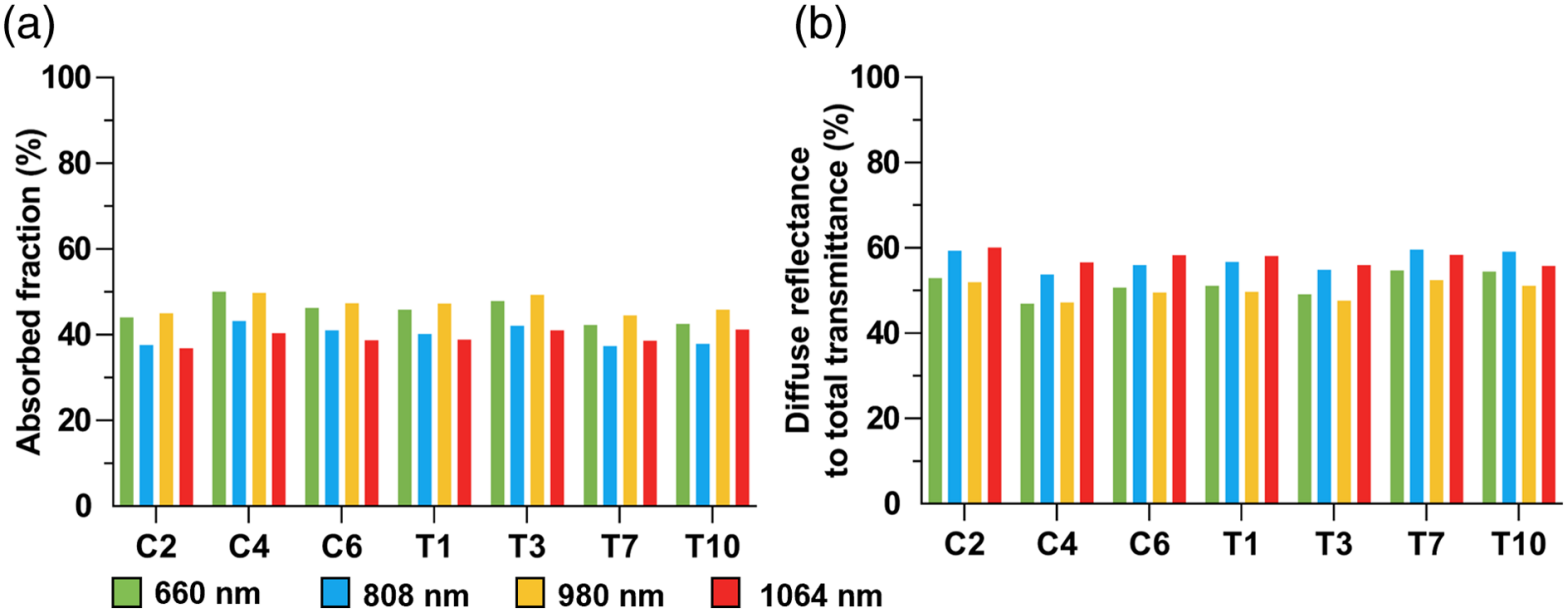
Absorbed fraction and diffuse reflectance, and total transmittance ratio. (a) Absorbed fraction, (b) diffuse reflectance, and total transmittance ratio.

When photons interact with biological tissues, they may be absorbed—converting energy into heat— or undergo two other additional processes: specular reflection, where photons are reflected at the tissue surface without entering, and diffuse reflection, where photons scatter multiple times within the tissue and are redirected back to the surface. Using the Monte Carlo simulation, we quantified specular reflectance, absorbed fraction, diffuse reflectance, and total transmittance for light at four wavelengths across seven SCI injury segments. The relative proportions of these three processes were then calculated and analyzed (Fig. 7).

The amount of light absorbed by biological tissues—contributing to thermal effects—varied only slightly across the seven spinal segments. However, modest differences were observed among the four wavelengths, with absorption varying by ∼50%. Specifically, the absorbed fraction of 660 nm light was generally slightly higher than that of 980 nm, and both were substantially greater than those of 808 and 1064 nm, which exhibited similar absorption levels. By contrast, diffuse reflectance and total transmittance together accounted for the majority of photon outcomes, typically exceeding 50% of the total photon interaction. These values were slightly higher in the thoracic spine compared with the cervical spine and were generally greater at 808 and 1064 nm than at 660 and 980 nm. Notably, the specular reflection—which was not included in the comparative bar plots—remained consistent at 3.01% across all models. This consistency is attributed to the fact that specular reflection depends on the refractive index mismatch at the interface between two media; in this study, light always entered from air to skin, keeping the reflectance consistent regardless of wavelength or spinal segment.

## 4 Discussion

In this study, based on the SD rat anatomy atlas, we constructed voxelized models (voxel size: $0.01\times 0.01\times 0.01\;{\mathrm{cm}}^{3}$) of the cervical spine (C2, C4, and C6) and thoracic spine (T1, T3, T7, and T10). We used the MCVM to simulate photon transmission from a Gaussian light source with four wavelengths (660, 808, 980, 1064 nm) and an intensity of 1 W, introduced from the skin surface to the injury site along the sagittal axis. The results indicated that cervical spine injuries may benefit more from light therapy than thoracic spine injuries, and for spinal cord injuries, the best treatment effect may be achieved with 1064 nm light. This study provides insights into model selection and treatment parameters for animal experiments on PBM for SCI.

We plotted the photon distribution curves along the sagittal axis of the injury center, with tissue types as the background, and pseudo-color images with the cross-sectional model of the injury center, to illustrate the depth and range of light propagation. To compare the photobiological effects of different wavelengths on tissues at various injury sites, we generated bar charts to show the total photon fluence in each tissue. We then calculated the proportion of photon fluence between tissues and created a series of donut diagrams. We also compiled and compared the light absorption in different tissues to characterize the thermal effects produced when light interacts with tissues. The reflection, absorption, and scattering of photons in tissues were also quantified, and their respective ratios were calculated. The simulation results indicated that in the entire spinal segment, light fluence in the cervical spinal (C2, C4, and C6) was greater than in the thoracic spine, whereas light absorption was higher in the thoracic vertebrae. This suggests that during PBM, photons at the cervical spine may induce more photobiological effects in the spinal cord, whereas those at the thoracic spine may induce more photothermal effects. Thus, light therapy for cervical spine injuries may be more effective than for thoracic spine injuries. Among the four wavelengths used in PBM, 1064 nm light showed greater penetration depth and photon fluence than 980, 808, and 660 nm. This suggests that in SCI light therapy, 1064 nm light may offer the best therapeutic effect.

A previous study employed a Monte Carlo simulation to evaluate light delivery in a 3D voxelized SCI rat model for PBMT with different irradiation parameters.^36^ They found that 980 nm light penetrated deeper into the model near the spine compared with 660 nm light, which is consistent with our findings. In addition to their study, we incorporated 1064 nm light for comparison. We also assessed the efficacy of different wavelengths at various spinal segments during PBM as spinal cord injuries can occur at any level, and the distance between the spinal cord and the skin differs significantly in the cervical and thoracic spine. Furthermore, previous studies^42^^,^^43^ compared light transmission at four wavelengths (808, 915, 975, and 1064 nm) in dog corpses. Consistent with these results, in the thoracic spine, the tissue irradiance at 1064 nm was notably greater than at the other three wavelengths. In addition, the optical clearing method can be used to enhance light penetration into tissues at these wavelengths. Briefly, hemoglobin acts as an optical clearing agent by making blood more transparent to light, aligning the refractive indices of blood plasma and erythrocytes.^44^ Recent studies have demonstrated that this method works in soft tissue when hemoglobin or whole blood is injected into the muscle tissue of rats, creating a hematoma and reducing light scattering in the NIR range within the hematoma area.^45^ This approach facilitates deeper photon penetration to the SCI site, thus enhancing the effectiveness of light therapy.

The results of this study suggested that cervical SCI may benefit more from light therapy than thoracic SCI, although there are relatively few animal models of cervical spine injury.^22^^,^^46^^–^^49^ This may be due to the increased difficulty of modeling cervical spine injuries and the potentially worse prognosis of cervical SCI, which poses significant challenges for phototherapy animal experiments. It is important to note that the MCVM can only simulate the specular reflection, absorption, or scattering of photons in tissues and infer the number and distribution of photons that generate photobiological and photothermal effects. However, it cannot compare different treatment parameters or models based on the actual mechanism of photon-tissue interaction.

As one of the neuromodulation technologies that regulates nerve function in SCI, PBM is non-invasive, low-cost, portable, and more adaptable to telemedicine compared with electrical stimulation, which requires multiple surgeries for implantation and adjustment.^7^ Although low-intensity pulsed ultrasound is also portable, its additional mechanical effects and potential adverse reactions remain controversial, making it less safe than PBM.^50^ Magnetic stimulation typically uses transcranial magnetic stimulation (TMS) to excite the motor cortex of SCI patients, generating induced currents in the cerebral cortex. This act on the upper motor neurons, inducing action potentials that are conducted along the descending conduction pathways, promoting axoplasmic transport, metabolism, and growth through repeated stimulation, and stimulating neural plasticity to facilitate compensatory recovery. As such, magnetic stimulation is generally effective only in the rehabilitation stage of SCI, whereas PBM can be used for treatment shortly after the injury occurs.^51^

## 5 Conclusion

Using Monte Carlo simulation and the SCI rat model, this study demonstrated that both wavelength and spinal cord segment significantly affect photon fluence distribution. The cervical spine may benefit more from light therapy than the thoracic spine due to its higher distribution to the spinal cord. The 1064 nm wavelength, in particular, exhibited superior penetration depth and photon fluence, making it potentially more effective for treating deeper spinal cords. These findings are essential for optimizing PBM treatments aimed at specific spinal levels. The detailed analysis of photon fluence distribution offers valuable insights into the optimal wavelengths and treatment parameters for effective photobiomodulation in SCI models.

## Supplementary Material

10.1117/1.JBO.30.S2. S23907.s01

## Data Availability

Code and data underlying the results presented in this paper are not publicly available at this time but may be obtained from the authors upon reasonable request at liting@bme.cams.cn
