# Supplementary material for: Effects of wavelength and spinal cord injury segment on photobiomodulation efficacy: Monte Carlo simulation in a rat model
Source: J Biomed Opt. 2025 Aug 4;30(Suppl 2):S23907. doi: 10.1117/1.JBO.30.S2.S23907 (PMC12322598; doi:10.1117/1.JBO.30.S2.S23907)
Supplement: Supplementary file 1 [file JBO_030_S23907_SD001.pdf]

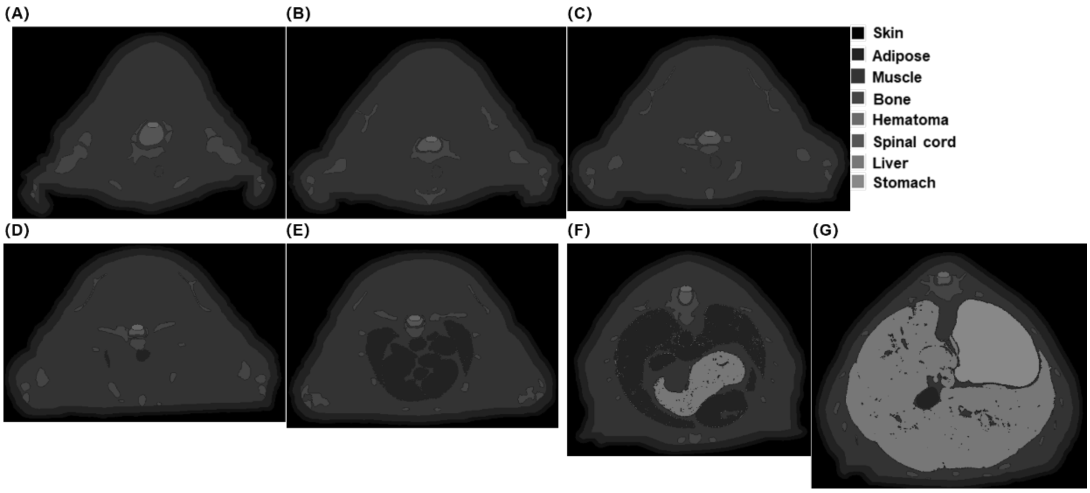

Supplementary figure 1. Grayscale images of seven injury centers after voxelization models were created.
